# A Colon Cancer Organoid-on-a-Chip Model for In Vitro Therapy Assessment

**DOI:** 10.3390/ijms27146427

**Published:** 2026-07-20

**Authors:** Luis G. Valle, Luis Ortega, Mariafe Laguna, Miguel Holgado

**Affiliations:** 1 Optics, Photonics and Biophotonics Group, Centre for Biomedical Technology, Campus de Montegancedo, Universidad Politécnica de Madrid, 28223 Madrid, Spain; luis.gvalle@upm.es; 2 Group of Organ and Tissue on-a-chip and In-Vitro Detection, Health Research Institute of the Hospital Clínico San Carlos, IdISSC, C/Profesor Martín Lagos s/n, 4^a^ Planta Sur, 28040 Madrid, Spain; 3 Biobank of Health Research Institute of the Hospital Clínico San Carlos, IdISSC, C/Profesor Martín Lagos s/n, 28040 Madrid, Spain; luis.ortega@salud.madrid.org; 4 Applied Physics and Materials Engineering Department, Escuela Técnica Superior de Ingenieros Industriales, Universidad Politécnica de Madrid, C/José Gutierrez Abascal, 2, 28006 Madrid, Spain

**Keywords:** colon cancer, organoids, organ-on-a-chips, in vitro testing, drug testing, 5-fluorouracil and oxaliplatin

## Abstract

Colon cancer is one of the leading causes of death, requiring advanced therapies that need models for developing new drugs. Conventional cell culture models do not accurately and precisely reproduce the complexity of the tumor microenvironment, limiting their usefulness in research and therapy development. To address this weakness, patient-derived organoids have emerged as promising in vitro models. The implementation of these organoid-based models into more physiologically relevant systems is expected to improve their clinical relevance. Thus, integrating these organoids into microfluidic chips acting as bioreactors will likely improve the predictive response of therapies in personalized medicine. In this article, we report the development of a new colon cancer organoid-on-a-chip model that enables the in vitro culture of patient-derived colon cancer organoids under continuous culture media flow. We demonstrate how the developed organoids were derived from tumor biopsies of patients with colorectal cancer, expanded in standard three-dimensional (3D) culture, and cultured inside the microfluidic chips. The microfluidic chip chambers are designed to house organoids in a controlled environment, allowing the injection of therapies and monitoring by optical microscopy in real time. The in vitro therapies tested were a combination of drugs based on 5-fluorouracil and oxaliplatin at different concentrations. As a result, we demonstrate for the first time that this model proves the capability of this technology for in vitro testing colon cancer therapies.

## 1. Introduction

Cancer is one of the most significant diseases occurring worldwide. Among the most common cancers, colorectal cancer ranks second or third as a cause of death; and as a result of an aging population, it is expected to increase to 2.5 million new cases by 2035 [1,2,3,4,5]. The development of new cancer drugs has high failure rates in clinical trials due to various factors such as preclinical models that do not provide adequate information on efficacy or toxicity [6]. In addition, tumors exhibit significant molecular and cellular heterogeneity between patients, which is a barrier to effective patient treatment. This requires the use of preclinical models that replicate human biological tumors and enable personalized cancer therapy [7,8]. On the other hand, neither animal models, which are deficient for human cancer because they do not present native tissue and the human tumor microenvironment, nor conventional bidimensional (2D) cultures reproduce the cellular tissue of living organs [9,10].

Microfluidics allows the development of platforms that provide a controlled environment for studying primary tissue samples with the appropriate physiological characteristics of the tissues. The combination of 3D tissues with microfluidic chip technology constitutes organ-on-chip (OoC) systems. OoC technology is a promising technological tool in cancer research as an alternative to traditional animal and cell culture models, offering advantages such as micro-nanoscale fluid handling, the use of small sample quantities, and low manufacturing costs [11]. Therefore, OoC technology allows for fluid control, concentration gradient control, and oxygen rate assessment, among others, leading to the possibility of efficiently evaluating alternative therapies in cancer treatments. In addition, these systems allow in vitro testing of organ models, real time monitoring and injection of controlled therapies [12]. Organ chips have been developed mimicking multiple types of organs, and good examples can be found in the scientific literature such as the lung [13], liver [13], kidney [14], pancreas [15], and heart [16], among others.

An organoid is a 3D cellular complex formed by cells differentiated from stem cells cultured in vitro. The use of organoids in microfluidic devices permits the modeling of a 3D culture with organ architecture and functional characteristics more similar to in vivo systems [17]. Organoids obtained from induced pluripotent stem cells (iPSCs) exhibit high cellular integrity and diversity of cell types. Organoids can also replicate the histological and genetic characteristics of the corresponding tumors allowing for a more representative and clinically relevant study of the evaluation for different therapies [18,19,20]. Current applications of tumor organoids are based on drug discovery and screening, as well as response prediction in personalized medicine [21,22,23]. Furthermore, the use of patient-derived organoids is a reliable predictive model because these 3D structures obtained from cancer stem cells represent the genome and phenotype of the tumor, making them highly suitable for drug studies [24,25,26,27].

By integrating three-dimensional tumor organoids into these microfluidic circuits, researchers have succeeded in recreating dynamic phenomena, overcoming the limitations of animal models and traditional static cultures to accelerate the discovery of new anticancer therapies [28]. This capability has made it possible, for example, to develop specific models of vascularized lung tumors that accurately mimic tumor infiltration into blood vessels, providing a highly sensitive platform for evaluating the actual efficacy of anti-invasive agents [29], as well as the opportunity to study the interaction between the microbiota and colon cancer, a critical factor in the development of this type of cancer [30]. Therefore, the integration of microfluidic chips with cancer research not only refines our understanding of tumor progression but also paves the way for precision preclinical evaluation.

Therapies for the treatment of patients with colorectal cancer are very broad, and there is great heterogeneity in responses to treatment as a whole. For this reason, it is necessary to predict and assess in vitro which treatment a patient is most likely to respond to, serving as a basis for personalized therapy in cancer treatment. Patients often receive drugs that are ineffective for their disease, as well as the side effects that this produces [28,29].

Chemotherapy is one of the mainstays of colon cancer treatment and is used to destroy cancer cells or prevent them from continuing to multiply. Chemotherapy regimens usually combine different drugs and are adjusted according to the stage of the cancer, the characteristics of the tumor, and the patient’s overall condition. Although chemotherapy can cause side effects, these are usually manageable, and the benefits of treatment have been shown to improve survival and quality of life for many patients. Initially, the development of therapies for colon cancer began with 5-fluorouracil (5-FU), used mainly in combination with leucovorin, which produced an increase in overall survival. Subsequently, another drug incorporated at that time is oxaliplatin, which, although not very effective as a single agent, has a synergistic effect when used in combination with 5-FU [30,31,32,33]. In 2000, the use of FOLFOX, a combination of oxaliplatin, leucovorin, and 5-FU, was published, and it was observed that the use of this treatment improved the prognosis [34,35,36,37].

In this work, we demonstrate the integration of patient-derived colorectal cancer organoids into a microfluidic platform, which enables the evaluation of cell proliferation and drug efficacy through optical microscopy to observe the organoids’ morphology, and fluorescence microscopy to assess expressed biomarkers under dynamic culture conditions. Specifically, the organoids were obtained from tumor biopsies of patients with colorectal cancer, simultaneously evaluating non-tumor samples from the same patient both in culture plates and in a microfluidic platform developed ad hoc for this application. In addition, different concentrations of drugs widely used in the treatment of this cancer were considered and evaluated, demonstrating the potential of using this colon cancer organoid-on-a-chip model for personalized medicine therapies and for the discovery and assessment of new drugs.

## 2. Results

### 2.1. Development of the Microfluidic Chip and Implementation of the Microfluidic System

**Figure 1:**
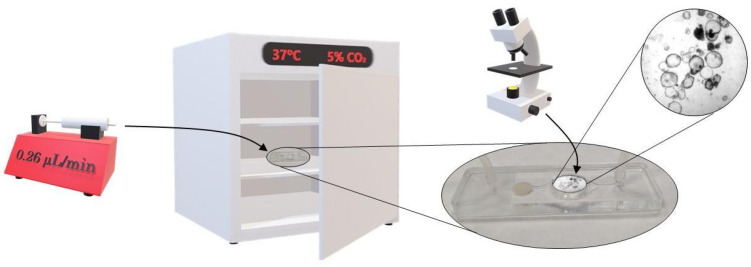
Microfluidic setup used during the drug screening assay: on the left is the pump, and on the right is the microfluidic chip in the incubator next to the Eppendorf tube.

For this study, a new microfluidic chip was designed and integrated into a microfluidic platform, resulting in a system capable of continuously supplying fresh culture medium to the sample, thereby mimicking physiological conditions. This chip consists of a chamber and a pre-chamber; the sample is cultured in the chamber, and the pre-chamber is designed to allow both drugs and fluorescent markers to be injected through a Polytetrafluoroethylene (PTFE) plug. These chambers are connected by a micro-fluidic channel. A pump is responsible for delivering the desired flow rate to the system via a tube that connects the syringe attached to the pump to the microfluidic chip. Finally, a tube carries the used medium to an Eppendorf tube (Figure 1). The microfluidic system was evaluated to ensure that it remained leak-proof throughout the entire experimentation process.

### 2.2. Derivation and Temporal Behavior of Organoids from Colorectal Cancer Biopsies in Culture Plates

**Figure 2:**
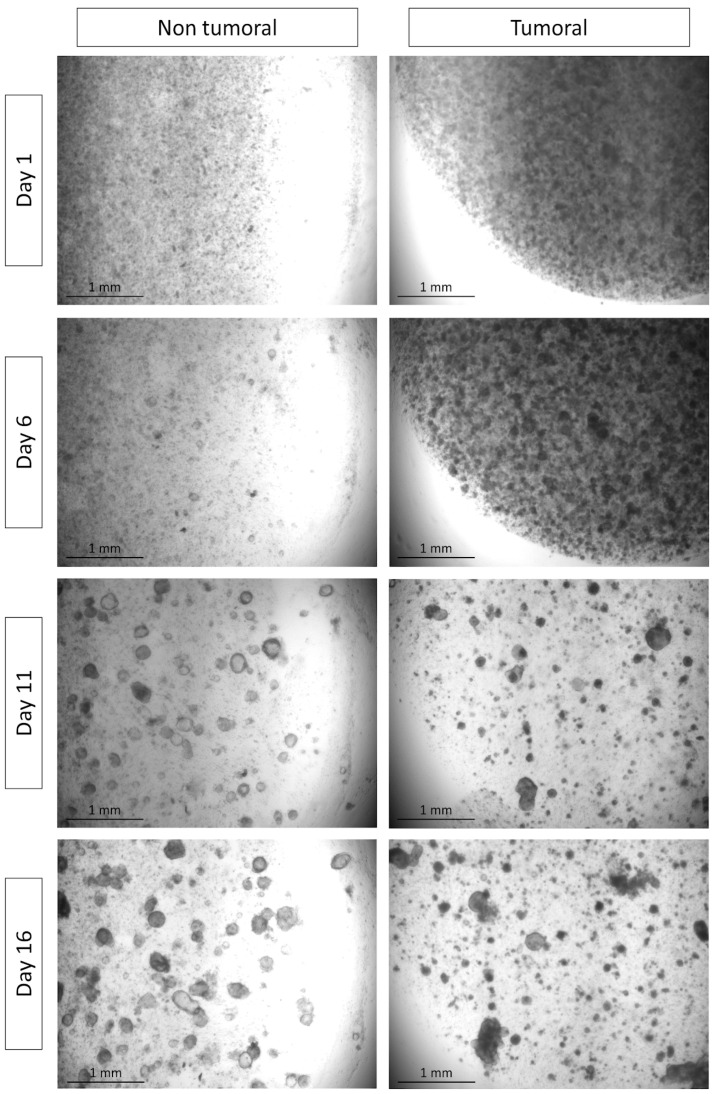
Images of a culture plate in which digested tumor tissue and non-tumor tissue have been cultured for 16 days.

First, tumor and non-tumor tissues are digested as described in the materials and methods section of this article, and organoids are obtained after approximately ten days (Figure 2). Once it becomes apparent that the matrix is becoming saturated with cells due to growth, the organoids are dissociated from the matrix and expanded into new wells. In the case of tumor organoids, they are transferred to a new matrix after one week, and in the case of non-tumor organoids, after two weeks. The organoids were monitored and analyzed using a Leica DMI3000 B inverted microscope equipped with a DFC 340 FX digital camera (Leica Microsystems, Wetzlar, Germany).

Longitudinal evolution of the sample with tumor cells (see Figure 2) reveals a clear progression in the increase in tumor cells over time. In the early stages (day 1), small and poorly defined structures are observed. From day six onwards, a notable increase in both the size and density of the structures is observed, accompanied by greater contrast, suggesting active processes of tumor growth. The average diameter of the organoids at that time was approximately 60 μm. The intermediate stage (day 11) shows pronounced coalescence, with irregularly shaped clusters occupying a larger fraction of the surface, and a wide range of sizes among the organoids, with an average size of 105 μm. In the later stages (day 16), it can be seen that the system contains large heterogeneous clusters, as well as smaller organoids, resulting in an average size of 175 μm. This observed temporal evolution and sustained growth demonstrates that the protocol used for organoid generation from biopsy-derived samples is effective, while also confirming the viability and long-term survival of the organoids under the established culture conditions.

The temporal analysis of the non-tumor sample (see Figure 2) demonstrates a progressive evolution during the culture period. The average diameter of the organoids was approximately 55 μm on day 6 (roughly 9.2 µm/day), increasing to 110 μm on day 11 (around 11 µm/day) and reaching about 200 μm on day 16 (18 µm/day). This gradual increase in size corroborates the efficacy of the applied culture conditions in supporting the growth, differentiation, and structural organization of cells derived from the non-tumor biopsy.

**Figure 3:**
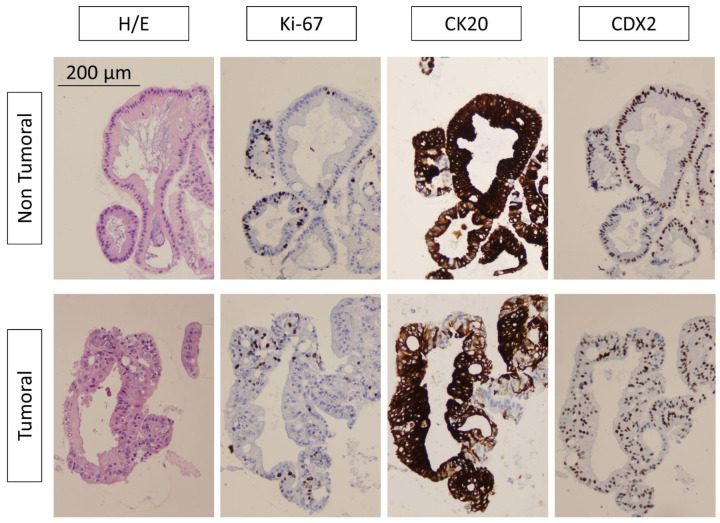
Characterization of tumor and non-tumor organoids using histology. From left to right: hematoxylin–eosin staining (H/E), immunohistochemistry with anti-Ki-67 antibody, immunohistochemistry with anti-CK20 antibody, and immunohistochemistry with anti-CDX2 antibody chips using bright-field microscopy. Hematoxylin stains mainly the cell nucleus dark purple; eosin stains the cytoplasm of the cells pink; and the antibodies used in immunohistochemistry are visible in dark brown.

Once the organoids had grown, one of the wells was fixed and characterized using immunohistochemistry. Hematoxylin–eosin (H/E) staining was used, revealing that the structure of the non-tumorous organoids is more organized than that of the tumorous ones, in which irregular nuclei are observed, and the characteristic cylindrical structure of intestinal tissue is absent, as can be seen in Figure 3. In addition, three antibodies targeting three antigens were employed:

- Ki-67, a nuclear marker that confirms the presence of dividing cells.
- CK20, a marker for colon epithelial cells, confirming differentiation.
- CDX2, a nuclear marker required for differentiation into enterocytes.

The Ki-67 protein is present during all active phases of the cell cycle (G1, S, G2, and mitosis), but is absent in resting (quiescent) cells (G0). As can be seen in Figure 3, Ki-67 is expressed in both tumor and non-tumor organoids, confirming that the culture medium promotes cell division.

Furthermore, the high expression of CDX2 and CDK20 shown in Figure 3 confirms that colon-derived organoids are forming and that these organoids contain cells differentiated into enterocytes.

### 2.3. Comparative Assessment Between Organoids on the Microfluidic Chip and on the Standard Culture Plates

After an additional week of culture, the organoids were dissociated and subsequently seeded onto microfluidic chips, as well as placed onto standard culture plates. This was done in order to test drugs on the organoids while supplying them with a continuous culture medium, and while carrying out the same experiment in parallel on a plate with static culture medium as a control. The organoids appeared morphologically similar under bright-field microscopy.

**Figure 4:**
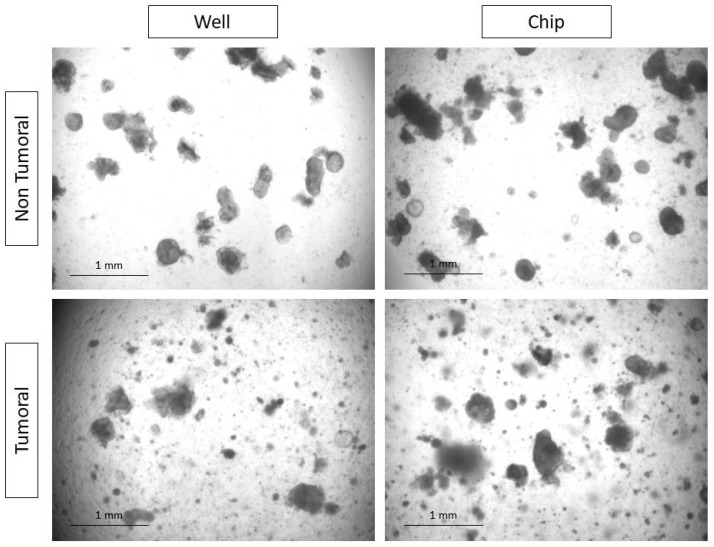
Comparison of organoids grown in plates and on chips by bright-field microscopy shows that there are no noticeable differences between chip-based and plate-based cultures; it can also be observed that the tumor organoids have a more irregular shape.

Following seeding, as illustrated in Figure 4, tumoral organoids cultured both on chips and on plates showed a higher density of small organoids around them in comparison with non-tumoral. As can be seen in the images, the chip allows organoids to be cultured just as effectively as on a culture plate. Therefore, although the organoids appeared morphologically similar under bright-field microscopy and no significant differences between chip-based and plate-based culture were observed, the microfluidic system stands out compared to conventional culture plates as it provides a more controlled and physiologically relevant environment, thanks to the continuous supply of culture medium and the greater stability of experimental conditions. This allows not only more efficient maintenance of organoid viability, but also a more accurate reproduction of their in vivo behavior, without compromising the culture efficiency observed in traditional plates.

### 2.4. Performance Evaluation of Therapies Using Bright-Field Microscopy

After seeding the organoids in the microfluidic chips, with the aim of observing in situ the evolution of the organoids, a combination of drugs commonly used in the treatment of this cancer was administered. Specifically, a combination of oxaliplatin and 5-fluorouracil at varying concentrations was infused through the fluidic channel; these drugs are widely used in the treatment of patients with colorectal cancer at the hospital where the biopsies are received. Three conditions were administered: A, no medication; B, low medication concentration (10 μM oxaliplatin and 10 μM fluorouracil); and C, high medication concentration (100 μM oxaliplatin and 100 μM fluorouracil). The drugs were allowed to act for four days to ensure complete perfusion from the syringe into the chip chamber.

5-FU is a thymidylate synthase inhibitor. This enzyme is responsible for synthesizing thymidine monophosphate through the methylation of uridine monophosphate; thymidine monophosphate is then phosphorylated to form thymidine triphosphate (dTTP), which is used in DNA replication. By inhibiting the action of this enzyme through the administration of 5-FU, a shortage of dTTP occurs, leading to the death of rapidly dividing cancer cells due to a lack of thymine. In addition, 5-FU can also affect RNA synthesis, as it can be converted within cells into 5-fluorouridine triphosphate (5FUTP), which can be incorporated into RNA in place of uridine. The incorporation of 5FUTP into precursor RNA disrupts RNA processing and maturation.

On the other hand, oxaliplatin has a cytotoxic effect because it is a platinum compound. Specifically, oxaliplatin forms cross-links both between DNA strands and within a single strand, which prevents DNA replication and transcription, leading to cell death.

**Figure 5:**
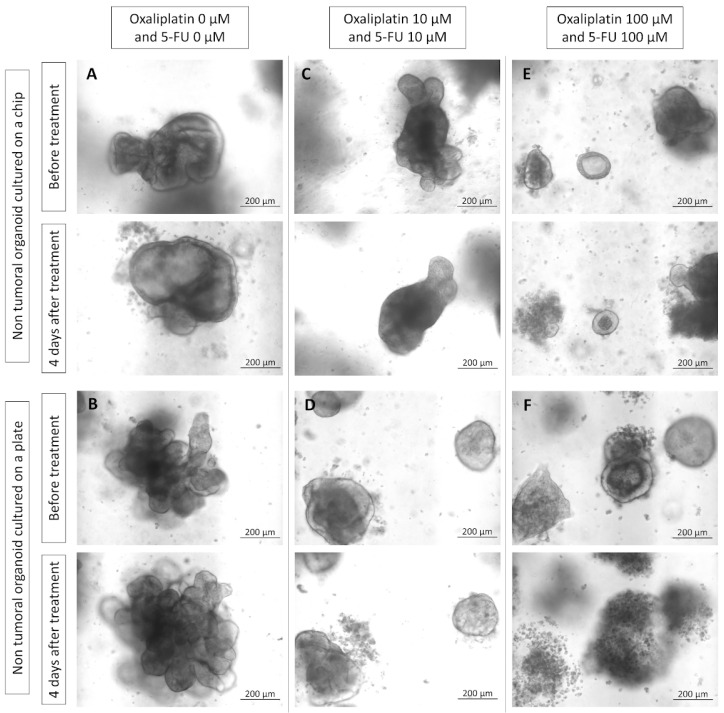
Evaluation of different concentrations of therapies in non-tumoral organoids cultured on chips ((**A**) no medication, (**C**) low medication concentration, and (**E**) high medication concentration) and in non-tumoral organoids cultured on plates ((**B**) no medication, (**D**) low medication concentration, and (**F**) high medication concentration) using bright-field microscopy.

**Figure 6:**
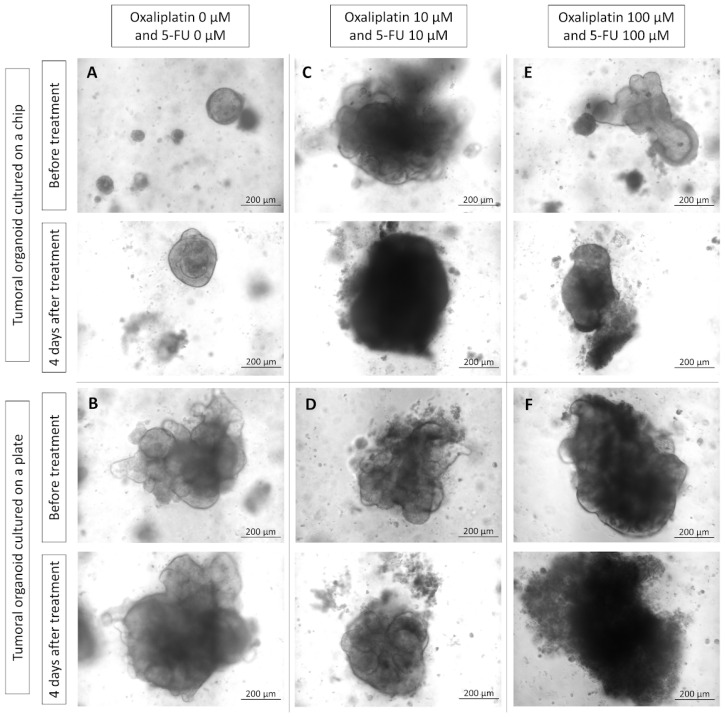
Evaluation of different concentrations of therapies in tumoral organoids cultured on chips ((**A**) no medication; (**C**) low medication concentration; and (**E**) high medication concentration) and in tumoral organoids cultured on plates ((**B**) no medication; (**D**) low medication concentration; and (**F**) high medication concentration) using bright-field microscopy.

In order to better assess the influence of these therapies, Figure 5 shows the temporal progression of the treatments for non-tumoral organoids in the microfluidic chips and cultured plates, respectively. Likewise, Figure 6 reflects this evolution of the treatments for tumoral organoids in the microfluidic chips and plates. Once the organoids had been treated with these drugs, we analyzed how their size changed. To do this, we measured the diameter of each organoid before and after treatment and calculated the relative change in size (%): (Ø_after_ − Ø_before_) × 100/Ø_before_.

**Figure 7:**
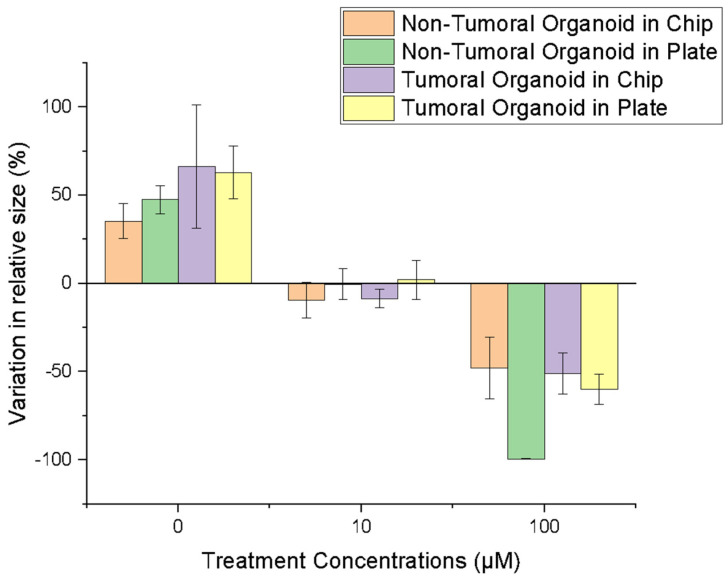
Evaluation of organoid relative change in size after culturing non-tumoral organoids in chip (orange) and in plate (green), and tumoral organoids in chip (purple) and in plate (yellow), with the three drug concentrations (0, 10, and 100 μM).

It can be observed in Figure 5 (therapies in non-tumoral organoid in the microfluidic chip) that lower drug concentrations preserved the organoid morphology of the non-tumor organoids cultured within the microfluidic device, with a slight decrease in size observed in organoid C from Figure 5 compared to a slight growth in Figure 5A, in the absence of treatment. In contrast, higher concentrations induced structural disintegration (Figure 5E). These observations correlate with the calculation of the relative change in size of the organoids, which is shown in orange in Figure 7.

In addition, in Figure 5 (therapies in non-tumoral organoids in plates), a slight growth can be observed with a drug concentration of 10 μM (organoid D), as well as in the control (organoid B). In addition, complete cellular disintegration is observed in the culture plate (Organoid F in Figure 5) at a concentration of 100 μM oxaliplatin and 100 μM 5-FU. Figure 7 shows in green that these non-tumor organoids cultured in plates behave similarly to those cultured on a chip, except when the maximum drug concentration is used, in which case the organoids completely disintegrate; this may be due to their size.

Tumor organoids cultured in the microfluidic chips showed marked growth in the absence of pharmacological treatment (organoid A in Figure 6), reflecting their high proliferative capacity under favorable conditions. At low drug concentrations (10 μM), slight growth is also observed (organoid C in Figure 6), suggesting that this dose is insufficient to significantly inhibit tumor proliferation. However, when the concentration is increased to 100 μM of oxaliplatin and 5-FU, a notable reduction in organoid size and viability is observed (organoid E in Figure 6), demonstrating a clear cytotoxic effect and a more effective therapeutic response at higher doses. This correlates with the analysis of organoid size when calculating the relative change in size, shown in purple in Figure 7.

Finally, tumor organoids cultured in plates in the absence of drugs also showed significant growth (organoid B in Figure 6), indicating strong proliferative capacity under untreated conditions. In contrast, tumor organoids exposed to a low drug concentration of 10 μM (organoid D in Figure 6) did not exhibit growth, suggesting that this treatment level is sufficient to inhibit proliferation but not necessarily to induce strong cytotoxic effects. Finally, at a high concentration of 100 μM oxaliplatin and 5-FU, complete cellular disintegration is observed in organoid F in Figure 6, indicating a marked cytotoxic response and effective elimination of the tumor organoid structure under these treatment conditions. Figure 7 shows that the calculation of the relative change in size of the organoids corresponds to what was observed under an optical microscope in Figure 6.

### 2.5. Performance Analysis of Therapies with Fluorescence Biomarkers

**Figure 8:**
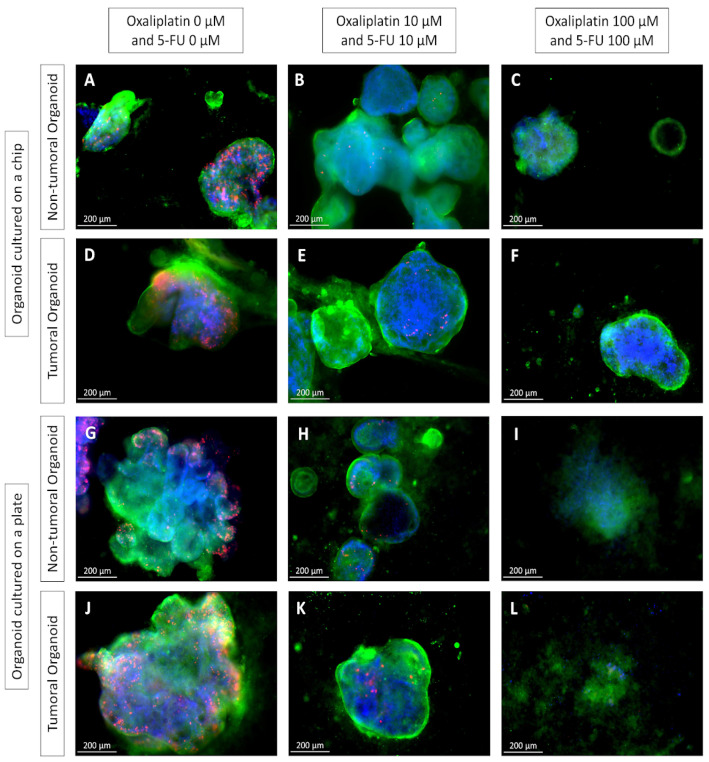
Organoid characterization using immunofluorescence: nuclei are shown in blue (Hoechst), the cell cytoskeleton in green (CK20), and dividing nuclei in red (Ki-67). Characterization of non-tumoral organoids ((**A**), no medication; (**B**), low medication concentration; and (**C**), high medication concentration) and tumoral organoids ((**D**), no medication; (**E**), low medication concentration; and (**F**), high medication concentration) cultured in microfluidic chips. Characterization of non-tumoral organoids ((**G**), no medication; (**H**), low medication concentration; and (**I**), high medication concentration) and tumoral organoids ((**J**), no medication; (**K**), low medication concentration; and (**L**), high medication concentration) cultured in plates.

Once the drugs were infused into the organoids, they were fixed and incubated with three fluorescent markers: blue for the nuclei (Hoechst), green for the cell cytoskeleton (CK20), and red for the dividing nuclei (Ki-67). The characterization with these markers can be seen in Figure 8, showing both non-tumoral and tumoral organoids in the microfluidic chip and in the culture plate.

As mentioned earlier, since a culture medium rich in growth factors was used, whose purpose is to keep the cells dividing, the Ki-67 antigen was selected as a marker. This protein is not present in quiescent cells, that is, those in the G0 phase, but its expression increases during all active phases of the cell cycle (G1, S, G2, and mitosis). Therefore, this marker allows us to determine whether the use of the two drugs causes a halt in cell division.

As can be observed in Figure 8, organoids A, D, G and J that have not received treatment (oxaliplatin 0 µM and 5-FU 0 µM) contained a large number of dividing nuclei (red dots). Once a small amount of drug is administered (oxaliplatin 10 µM and 5-FU 10 µM), it is difficult to see dividing nuclei in organoids B, E, H and K from Figure 8, although some are present. However, it should be noted that chips with a higher concentration of oxaliplatin 100 µM and 5-FU 100 µM clearly showed dead organoids; in this case, the structure of the organoids C, F, I and L, which appears green, breaks down, and diffuse green patches become visible (Figure 8). In addition, smaller blue dots—smaller than the nuclei normally observed and not contained within these structures—can be seen, which could be clusters of nuclear material. Moreover, it can also be observed that there is no presence of the red marker of dividing nuclei in the microfluidic chips; this also occurs in these organoids cultured on plates (Figure 8).

As a result, these observations indicate that an increase in the amount of drug administered is associated with a progressive reduction in the number of dividing nuclei, as indicated by the decreased presence of red-stained nuclei. These observations also suggest a dose-dependent inhibitory effect of the drug on nuclear division, which is clearly visible in the organoids cultured within the microfluidic chip and therefore demonstrates that this device is a feasible platform for carrying out detailed therapeutic studies in colon cancer.

## 3. Discussion

In this work, we developed and validated a colon cancer organoid-on-a-chip model from patient-derived tumoral and non-tumoral colon organoid cultures. We have also evaluated the potential of these microfluidic-based chip platforms for drug testing and biomarker detection. Organoids were generated using a protocol optimized for their production through the culture of crypts isolated from human biopsies; furthermore, their growth over time was also assessed in a plate assay. It is relevant to highlight the demonstrated feasibility in this scientific report to integrate colorectal cancer organoids into a dynamic microfluidic environment, as this system preserves what is observed in conventional plate cultures, offering additional physiological relevance thanks to continuous perfusion and controlled drug delivery.

One of the main limitations of conventional plate cultures is their inability to replicate tumor microenvironment aspects, such as nutrient gradients, waste removal, and continuous exposure to drugs. By using the microfluidic chip presented in this paper, these limitations are overcome thanks to the input of a continuous flow of culture medium that more closely mimics in vivo conditions. It is important to note that the flow rate used in the chips was adjusted to match the actual renewal of the culture medium used in the 24-well plates, allowing for a direct comparison between the two systems. Under these conditions, organoids cultured on chips maintained their structural integrity and viability, indicating that the microfluidic environment does not induce additional stress compared to static cultures.

A combination of therapies commonly administered to patients with this type of cancer was used, employing three different concentrations: a control condition, an intermediate concentration, and a high concentration in order to observe differences in the response. The response of tumor and non-tumor organoids to oxaliplatin and 5-FU supports the suitability of this platform for therapeutic testing. In non-tumor and tumor organoids, low drug concentrations (0 and 10 µM) preserved the architecture of the organoids, while higher concentrations (100 µM) caused cell death, which can also be observed in the literature [38]. The concordance between chip-based and plate-based cultures suggests that the microfluidic system reliably reproduces the drug responses observed in standard assays.

The concentrations of oxaliplatin (10 and 100 μM) and 5-fluorouracil (10 and 100 μM) used in this study were selected in accordance with previously published protocols for organoid models derived from patients with colorectal cancer, aiming to facilitate comparison with established preclinical studies.

However, to put these concentrations into clinical context, it should be noted that pharmacokinetic studies show that, following intravenous administration of oxaliplatin, peak plasma concentrations of approximately 4.66 ± 1.38 μg/mL are reached, equivalent to about 11.7 ± 3.5 μM. In the case of 5-fluorouracil, the authors observed high variability in plasma concentrations during the infusion, with values ranging from 23.9 to 533.8 ng/mL, which is equivalent to 0.18–4.1 μM [39]. Consequently, the 10 μM concentration used in this study falls within the range of maximum plasma concentrations reported in patients, whereas the 100 μM concentration represents a higher level of exposure commonly used in in vitro studies to evaluate the cellular response to high doses.

Although a culture medium containing growth factors that promote cell division is being used, we can see that the use of 5-FU and oxaliplatin halts cell division. Immunofluorescence analysis confirms the morphological observations. The absence of Ki-67-positive nuclei is observed in organoids treated with higher concentrations of the drug, confirming effective inhibition of proliferation, while these proliferative markers are observed in untreated conditions or at low doses, indicating that cellular activity is preserved, although when using low-concentration medication, the number of nuclei observed is very low.

Finally, this study focuses on molecular and cellular mechanisms involved in colorectal cancer using advanced organoid-on-a-chip technology. The work integrates molecular biology, cancer therapeutics, biomarker detection, and drug-response analysis through patient-derived tumor and non-tumor organoids cultured in a microfluidic environment. In addition, the evaluation of chemotherapeutic agents such as 5-FU and oxaliplatin, together with the analysis of proliferation markers like Ki-67, highlights the molecular and translational relevance of the study. Therefore, we consider that the manuscript is of high interest for the scientific constituency in molecular medicine, cancer biology, biomarker research, and innovative experimental platforms for therapeutic testing.

## 4. Materials and Methods

### 4.1. Microfluidic Chip Fabrication

**Figure 9:**
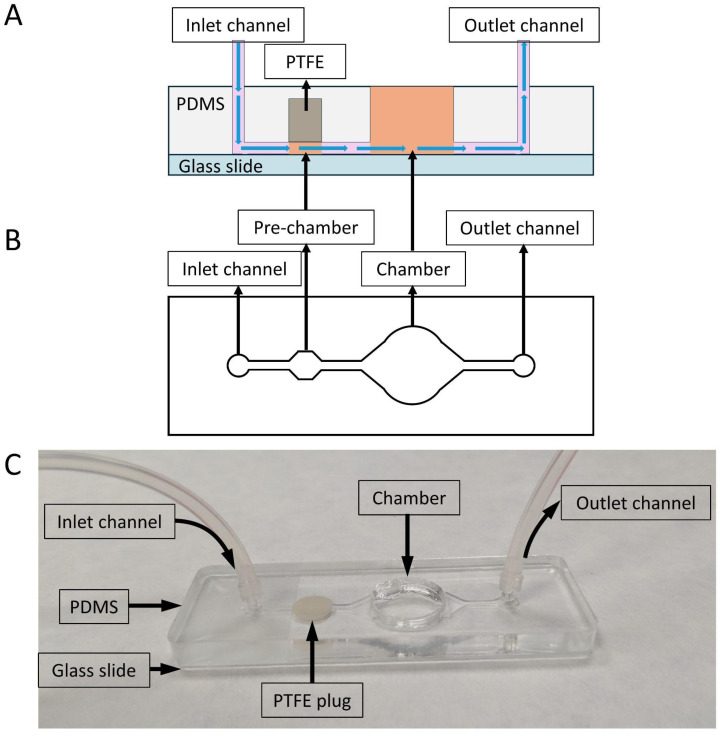
(**A**) Cross-section of the Microfluidic chip. (**B**) Top view of the Microfluidic chip. (**C**) Photograph showing the parts of the Microfluidic chip. The blue arrows indicate the direction of flow; the red area indicate the locations of the prechamber and the chamber; and the gray area indicate the location of the PTFE plug.

The microfluidic chips are made of a Polydimethylsiloxane (PDMS) block, which is attached to a glass slide. This PDMS block is manufactured by mixing the monomers with 10% crosslinker to initiate polymerization, after which it is deposited onto a template mold. Once the PDMS has been deposited, a PTFE plug is added to the pre-chamber so that any type of compound can be injected. After 1 h at 90 °C, the PDMS blocks solidify. The mold determines the microfluidic structure of the chip. Figure 9 shows the design of the chip consisting of a chamber where the organoid will be cultured, a pre-chamber, which is designed to allow a compound to be injected into the chip during incubation, and inlet and outlet channels, as well as a continuous fluidic channel that connects the inlet and outlet channels of the culture medium and passes through the pre-chamber and the chamber.

The chip consists of a 1.2 mm wide fluidic channel that carries the culture medium from the inlet channel to the outlet channel (50 mm), passing through the prechamber and the chamber. The chamber consists of a circle with a diameter of 15 mm. The dimensions of the glass are 25 mm in height and 70 mm in length.

To attach the PDMS block to the glass slide, an oxygen plasma treatment is performed for 1 min at 180 W. Next, pressure is applied, and they are placed in an oven at 80 °C for one hour. Once the sample is placed, the chip is covered. Finally, once the chip is assembled, one tube is connected to the inlet channel and another to the outlet channel. The inlet tube is connected to a syringe with culture medium, which is placed in a perfusion pump, while the outlet tube is connected to an Eppendorf tube that will collect the culture medium (see microfluidic set-up in Figure 1).

Although it is essential to take shear stress into account in many organ-on-chip systems, since the cells are directly attached to the channel or primary tissue is being cultured, it can affect viability. However, in this study, organoids are embedded in a matrix, so they are physically separated from the flow, and nutrients and drugs reach them by diffusion. Therefore, we believe that the shear stress generated by the flow does not have a significant effect on the organoids; consequently, this parameter was not included in the analysis of the system.

### 4.2. Obtaining the Organoid Through Biopsies

Experiments were conducted in accordance with the tenets of the Declaration of Helsinki for biomedical research involving human tissue. Samples were collected from colon cancer patients after obtaining their written informed consent, and the experiments were approved by the Ethics Committee (CEIm) of the Hospital Clínico San Carlos of Madrid, Spain. Two types of organoids derived from colorectal tissue were used in this study: tumor tissue organoids and non-tumor tissue organoids obtained from tissue adjacent to the tumor.

This study was designed as a proof-of-concept analysis to evaluate the feasibility of the microfluidic platform that was developed. Before conducting the study described in this scientific report, the experimental protocol was optimized through preliminary studies using samples from different patients, which allowed us to establish the conditions for isolation, culture, and analysis used in this study. Once the methodology was optimized, a patient was selected for whom both tumor and non-tumor tissue were available to perform the comparison presented in this manuscript. Although these results demonstrate the feasibility of the platform, future studies involving a larger number of patients will permit the validation of its reproducibility and its application in a broader clinical context.

The biopsies obtained were washed with cold PBS and the supernatant was removed. The biopsies were then physically digested with scissors and a scalpel, followed by a digestion solution (Gentle Cell Dissociation Reagent, Stemcell, Vancouver, BC, Canada, ref.: 100-0485). From this point on, both the tips and tubes were coated with 1% Bovine serum albumin (BSA) in Dulbecco’s Modified Eagle’s Medium/Ham’s F-12 Nutrient Mixture (DMEM/F12. Merck, Darmstadt, Germany, ref.: D8437). After incubating the biopsies in the digestion solution in motion for 30 min, they are shaken vigorously with 1 mL of DMEM/F12 with 1% BSA, using a pipette to better remove the crypts from the tissue.

Subsequently, they are passed through a 70 µm filter, the number of crypts in 10 µL of the digestion is counted for both cases (tumor and non-tumor tissue), and the number of crypts in 1 mL is calculated. They are centrifuged at 290× *g* for 5 min and resuspended in DMEM/F12 culture medium. In the case of non-tumor tissue, 200 crypts are used for each well in which these organoids are to be cultured. Next, 25 µL of Matrigel 3D matrix (Corning™, New York, NY, USA, ref.: 356231) is added cold to 25 µL of crypts or 25 µL of tumoral cells in culture medium; then, they are placed in cultured plates in an incubator at 37 °C for 30 min to allow the matrix to solidify. Finally, once solid, Intesticult culture medium (Stemcell, ref.: 06010) with 1% penicillin/streptomycin is added. The culture medium is changed every two days. In addition, every 15 days, the organoids are separated from the matrix, centrifuged, and resuspended in cold medium together with new matrix to later seed them in a new culture plate. After 15 days, it can be observed that between 150 and 200 organoids are obtained per well, each with an approximate diameter of 200 μm. Following this culture, they were expanded into new wells, and after another 15 days, 50 organoids were used per experiment. This protocol is the one established by Stemcell Technologies for the IntestiCult™ culture medium.

**Figure 10:**
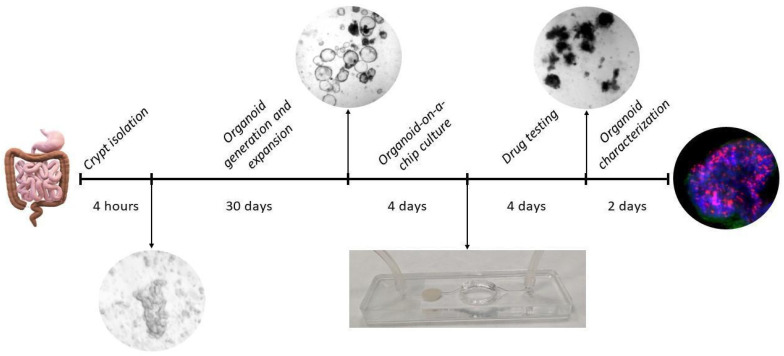
Time schedule for organoid generation and characterization.

Once the organoids have been grown on a plate for 30 days, they are separated from the matrix, centrifuged, and resuspended in a cold medium together with a new matrix. Next, 50 μL of the organoid solution, culture medium and matrix are seeded on each microfluidic chip, and a lid is placed on these chips; in turn, 50 μL of the solution is seeded in several wells of a P24 plate. The flow used in the chips is 0.26 μL/min, the equivalent of 0.75 mL of medium every two days, which is the amount used in the wells (Figure 10).

### 4.3. Monitoring by Optical Microscopy

Every two days, the organoids were systematically monitored and analyzed using a Leica DMI3000 B inverted microscope equipped with a DFC340 FX digital camera. During each observation session, high-resolution images were acquired from both the microfluidic chips and the wells of the culture plates in order to document organoid morphology, growth progression, and overall structural integrity throughout the experimental period.

### 4.4. Characterization by Immunohistochemistry

Once the organoids have been expanded, one of the wells is used to characterize the organoids. For this well, the culture medium is collected, and 500 μL of 4% paraformaldehyde (PFA) in Phosphate-Buffered Saline (PBS) is added at room temperature for 15 min.

After removing the fixing solution, the organoids were embedded in paraffin, cut into 4 μm thick sections, and mounted on slides. For hematoxylin–eosin staining, the slides were rinsed in xylene and then passed through decreasing concentrations of ethanol to deparaffinize and rehydrate them. Next, they were washed with water and stained, and finally dehydrated by reversing the alcohol concentration process before being covered with coverslips.

When antibodies were used, the sections were incubated in a humidifier in an antigen retrieval buffer with a pH specific to the antibody; they were then cooled and incubated in PBS containing blocking buffer. Subsequently, they were incubated with the corresponding primary antibody in blocking buffer. Afterward, the slides were incubated in 0.3% H_2_O_2_. The slides were then incubated with the corresponding HRP-conjugated secondary antibody. Finally, the slides were developed using the DAB-peroxidase (HRP) substrate kit and 3,3′-diaminobenzidine.

### 4.5. Insertion of Anti-Cancer Therapies

After incubating the organoids on microfluidic chips for 4 days, a mixture of two drugs used in the treatment of colon cancer, namely oxaliplatin and fluorouracil (5-FU), is supplied through the culture medium. There are 3 conditions:

- Condition A: 0 μM oxaliplatin together with 0 μM fluorouracil (5-FU). Because no medication is supplied, the organoids should continue to grow.
- Condition B: 10 μM oxaliplatin together with 10 μM fluorouracil (5-FU). According to the literature, at this drug concentration, the organoids stop growing [38].
- Condition C: 100 μM oxaliplatin combined with 100 μM fluorouracil (5-FU). In contrast, at this drug concentration, the organoids undergo a process of apoptosis.

These conditions are also added to wells to observe whether the chip and plate cultures are similar.

To quantify the effect of the treatments on organoid growth, three representative organoids from each experimental condition were selected, and their diameter was determined using image analysis. The results are expressed as the mean ± standard error.

### 4.6. Monitoring Using Fluorescent Biomarkers

After culturing organoids for 4 days with the drug mixture (or without it) in both 24-well plates and microfluidic chips, the culture medium is collected, and 500 μL of 4% PFA in PBS is added at room temperature for 15 min. After removing the fixing solution, each well/chip is washed three times with cold PBS, and 1 mL of a permeabilizing solution (PBS-BSA (1%)-Triton-X100 (0.1%)) is added for 60 min at room temperature. Next, each well/chip is washed three times with cold PBS, and rabbit anti-Ki-67 antibody (1:500, Thermofisher, Waltham, MA, USA, ref.: MA514520) and anti-CK20 (1:500, Agilent, Santa Clara, CA, USA, ref.: GA77761-2) are added and incubated overnight at 4 °C. Ki-67 is a nuclear protein that is related to cellular proliferation, and CK20 is a structural protein expressed in epithelial cells of the gastrointestinal tract. Then, each well/chip is washed three times with cold PBS and goat anti-rabbit AlexaFluor 568 antibody (1:1000, Thermofisher, ref.: A21207) and goat anti-mouse AlexaFluor 488 antibody (1:1000, Thermofisher, ref.: A11001) are incubated for 2 h at RT. Finally, all wells and chips are washed three times with cold PBS, and Hoechst stain (blue fluorescent dye that binds nuclear DNA, Deoxyribonucleic acid) is added to each well/chip. Once washed, the wells and each chip chamber are covered with coverslips, and images are obtained after 24–48 h. Images are captured using an immunofluorescence microscope at 10× magnification.

In this manner, and by means of the previously mentioned microscope, it is possible to quantify and compare the number of nuclei undergoing division under each experimental condition. This approach allows for the observation of a clear trend whereby increasing concentrations of the drug are associated with a progressive decrease in the number of dividing nuclei, indicating a concentration-dependent effect on cellular proliferation.

For each experimental condition, a separate microfluidic chip was used, on which approximately 50 organoids embedded in Matrigel were cultured. The immunofluorescence images presented here correspond to representative organoids from each condition and reflect the expression pattern consistently observed across the set of cultured organoids.

## 5. Conclusions

This study provides a significant proof of concept for the successful integration of patient-derived colorectal cancer organoids into a dynamic microfluidic organ-on-chip platform. The system maintains organoid viability and architecture under continuous perfusion and enables reproducible, dose-dependent evaluation of chemotherapeutic response through morphological and proliferation analyses.

This microfluidic system also demonstrates a decrease in cell division as the concentration of the drug increases, as revealed by immunofluorescence analysis, specifically by observing a decrease in Ki-67 expression in the nuclei of the organoids.

The concordance between chip and conventional plate cultures validates the reliability of the platform, while the dynamic environment enhances its physiological relevance. Although further validation in a larger patient cohort is required, and comparison with in vivo concentrations observed in patients with this in vitro model is needed, this work represents a promising step toward more predictive in vitro systems and future applications in personalized therapy for colorectal cancer.

## Data Availability

The original contributions presented in this study are included in the article. Further inquiries can be directed to the corresponding authors.
